# Cholesterol and Egg Intakes with Cardiometabolic and All-Cause Mortality among Chinese and Low-Income Black and White Americans

**DOI:** 10.3390/nu13062094

**Published:** 2021-06-19

**Authors:** Xiong-Fei Pan, Jae-Jeong Yang, Loren P. Lipworth, Xiao-Ou Shu, Hui Cai, Mark D. Steinwandel, William J. Blot, Wei Zheng, Danxia Yu

**Affiliations:** 1 Vanderbilt Epidemiology Center, Vanderbilt University Medical Center, Department of Medicine, Division of Epidemiology, Nashville, TN 37203, USA; xiongfei.pan@vumc.org (X.-F.P.); jae.j.yang@vumc.org (J.-J.Y.); loren.lipworth@vumc.org (L.P.L.); xiao-ou.shu@vanderbilt.edu (X.-O.S.); hui.cai@vumc.org (H.C.); william.j.blot@vumc.org (W.J.B.); wei.zheng@vanderbilt.edu (W.Z.); 2 International Epidemiology Field Station, Vanderbilt University Medical Center, Nashville, TN 37203, USA; mark.d.steinwandel@vumc.org

**Keywords:** dietary cholesterol, egg intake, cardiometabolic disease, mortality, prospective cohort study

## Abstract

We examined the associations of dietary cholesterol and egg intakes with cardiometabolic and all-cause mortality among Chinese and low-income Black and White Americans. Included were 47,789 Blacks, 20,360 Whites, and 134,280 Chinese aged 40–79 years at enrollment. Multivariable Cox models with restricted cubic splines were applied to estimate hazard ratios (HRs) and 95% confidence intervals (CIs) for mortality outcomes using intakes of 150 mg cholesterol/day and 1 egg/week as the references. Cholesterol intake showed a nonlinear association with increased all-cause mortality and a linear association with increased cardiometabolic mortality among Black Americans: HRs (95% CIs) associated with 300 and 600 mg/day vs. 150 mg/day were 1.07 (1.03–1.11) and 1.13 (1.05–1.21) for all-cause mortality (*P*-linearity = 0.04, *P*-nonlinearity = 0.002, and *P*-overall < 0.001) and 1.10 (1.03–1.16) and 1.21 (1.08–1.36) for cardiometabolic mortality (*P*-linearity = 0.007, *P*-nonlinearity = 0.07, and *P*-overall = 0.005). Null associations with all-cause or cardiometabolic mortality were noted for White Americans (*P*-linearity ≥ 0.13, *P*-nonlinearity ≥ 0.06, and *P*-overall ≥ 0.05 for both). Nonlinear inverse associations were observed among Chinese: HR (95% CI) for 300 vs. 150 mg/day was 0.94 (0.92–0.97) for all-cause mortality and 0.91 (0.87–0.95) for cardiometabolic mortality, but the inverse associations disappeared with cholesterol intake > 500 mg/day (*P*-linearity ≥ 0.12; *P*-nonlinearity ≤ 0.001; *P*-overall < 0.001 for both). Similarly, we observed a positive association of egg intake with all-cause mortality in Black Americans, but a null association in White Americans and a nonlinear inverse association in Chinese. In conclusion, the associations of cholesterol and egg intakes with cardiometabolic and all-cause mortality may differ across ethnicities who have different dietary patterns and cardiometabolic risk profiles. However, residual confounding remains possible.

## 1. Introduction

Cardiovascular disease (CVD) and diabetes are leading causes of death worldwide. The global age-standardized death rate from CVD and diabetes combined reached up to 250 per 100,000 people in 2017 [1]. Nutrition plays a substantial role in the development and progression of CVD and diabetes [2,3]. As hypercholesterolemia, which describes particularly high levels of low-density lipoprotein cholesterol, is an established risk factor for CVD, reducing dietary cholesterol intake has been a presumed effective strategy to prevent CVD and premature death over the last few decades. However, the 2015–2020 Dietary Guidelines for Americans removed the recommendation of limiting cholesterol intake to no more than 300 mg/day [4], as did other major guidelines for CVD prevention and control [5,6].

Eggs are a major source of dietary cholesterol and other nutrients such as high-quality protein and vitamins. Highly affordable and accessible, eggs are often recommended as part of a healthy diet [4]. However, due to their high cholesterol content (~186 mg in a large egg), the association between egg intake and risk of or mortality from CVD has long been debated. Several meta-analyses of observational studies did not find significant associations of up to 1 egg/day or dietary cholesterol intake with a risk of CVD [7,8,9,10,11]. However, a pooling analysis of six prospective studies of US populations in 2019 reported strong dose–response associations of both cholesterol and egg intakes with incident CVD and all-cause mortality [12], which was confirmed in two recent large studies including over 520,000 US men and women [13] and among over 96,000 US postmenopausal women [14]. In addition, a meta-analysis of 28 prospective studies in 2020 suggested potential population-specific associations between egg consumption and CVD risk: an inverse association was observed in Asian cohorts only, but not in US or European cohorts [11]. The suggested discrepant egg–CVD associations across populations may be due to differences in dietary patterns and cardiometabolic health status across sociodemographics and ethnicities [15,16]. It is thus important to conduct cross-population studies on this topic, particularly involving understudied populations such as Black Americans and low-income populations.

To this end, we examined the associations between the dietary intake of cholesterol and eggs with cardiometabolic (i.e., coronary heart disease (CHD), stroke, and diabetes) and all-cause mortality among low-income Black and White Americans from the Southern Community Cohort Study (SCCS) [17] and among Chinese adults from the Shanghai Women’s Health Study (SWHS) [18] and the Shanghai Men’s Health Study (SMHS) [19]. We also investigated potential heterogeneities in the associations in subpopulations defined by major CVD risk factors or preexisting conditions.

## 2. Materials and Methods

### 2.1. Study Population

The study population comprised participants from three large prospective cohort studies. The SCCS recruited a total of 84,735 primarily low-income Americans (~66% were Black Americans and >50% had a household income < 15,000 US dollars (USD)/year) aged 40–79 years in 12 southeastern US states between 2002 and 2009 [17]. The SWHS recruited 74,940 women aged 40–70 years between 1996 and 2000 [18], and the SMHS enrolled 61,480 men aged 40–74 years between 2002 and 2006 in Shanghai, China [19]. Informed consent was obtained from study participants at enrollment. In baseline surveys, each cohort collected information on sociodemographics, lifestyle and dietary factors, medical history, and anthropometrics. All study participants were followed up regularly according to the study protocols. The original cohort studies were approved by the Institutional Review Boards of Vanderbilt University Medical Center, Meharry Medical College, and/or the Shanghai Cancer Institute. For the current study, de-identified data were harmonized with the approval of the Data Use Committee of each cohort.

For study purposes, we excluded individuals who reported implausible total energy intake using predefined study-specific cut-off points (SCCS, <600 or >8000 kcal/day (i.e., approximate top and bottom 2.5%); SWHS, <500 or >3500 kcal/day and SMHS, <800 or >4200 kcal/day) based on previous recommendations [20,21] or had missing data on dietary cholesterol or eggs (*n* = 7167), or who had invalid data on vital status or follow-up time (*n* = 398). To minimize the potential influence of reverse causation, we further excluded 7378 participants who had a history of cancer and 338 who died or were lost to follow-up within the first year after the baseline survey. For the SCCS, we only included Black and White Americans because the sample size of other ethnic groups was very small (*n* = 3445). After these exclusions, the final study population included a total of 202,429 participants, comprised of 47,789 Black Americans, 20,360 White Americans, and 134,280 Chinese (Figure S1).

### 2.2. Assessment of Dietary Intakes

Dietary information was collected at baseline using food frequency questionnaires (FFQs), which comprised foods commonly consumed in respective study populations. The design, validity, and reproducibility of the three FFQs have been described elsewhere [20,22,23,24]. Briefly, the SCCS FFQ inquired about the consumption frequencies of 89 food items. Standard portion sizes were assigned using the ethnicity- and sex-specific portion sizes from the National Health and Nutrition Examination Survey (NHANES) and the United States Department of Agriculture Continuing Survey of Food Intakes [22,25,26]. Total energy and nutrient intakes were estimated using the United States Department of Agriculture Food Composition Databases [20]. The FFQs for SWHS and SMHS were similar and contained questions on consumption frequencies and quantities of 77 and 81 food items, respectively. Total energy and nutrient intakes for the two cohorts were calculated using the 2002 Chinese Food Composition Tables [27].

### 2.3. Outcome Ascertainment

According to the SCCS protocol, the vital status, death date, and underlying cause of death were ascertained via annual linkage of the cohort to the National Death Index and Social Security Administration mortality files. The current analysis in the SCCS included follow-up data through 31 December 2015. The SWHS and SMHS followed up study participants via an annual linkage to the Shanghai Vital Statistics Registry. The current analysis in the SWHS/SMHS used vital status data through 31 December 2016. Our primary outcomes were all-cause and cardiometabolic mortality, which included deaths due to any types of CVD and diabetes. Mortality from CHD, stroke, or diabetes was evaluated individually as secondary outcomes. The underlying causes of death were defined using the International Classification of Diseases, 9th and 10th Revision, and were coded as follows: 390–459 and I00–I99 for total CVD, 410–414 and I20–I25 for CHD, 430–438 and I60–I69 for stroke, and 249–250 and E10-E14 for diabetes.

### 2.4. Covariate Assessments

Baseline information of covariates was collected using cohort-specific questionnaires and were harmonized for the current study. Covariates included age (continuous), sex (men and women), education (less than high school, high school graduation, college education, and university degree or above), annual income (low, lower-middle, upper-middle, and high; for the SCCS: annual household income < 15,000 USD, ≥15,000 to <25,000 USD, ≥25,000 to <50,000 USD, and ≥50,000 USD; for the SWHS: annual household income < 10,000 Chinese Yuan (CNY), ≥10,000 to <20,000 CNY, ≥20,000 to <30,000 CNY, and ≥30,000 CNY; and for the SMHS: annual personal income < 6000 CNY, ≥6000 to <12000 CNY, ≥12,000 to <24,000 CNY, and ≥24,000 CNY), marital status (married and single/separated/divorced/widowed), total energy intake (kcal/day; continuous), smoking status (never, former, and current), smoking pack-years (continuous), alcohol consumption (none, moderate (>0 to ≤2 drinks per day in men or >0 to ≤1 drink per day in women), and heavy drinking (>2 drinks per day in men or >1 drink per day in women); 1 drink = 14 g ethanol), physical activity (ethnicity- and sex-specific tertiles of total metabolic equivalent hours per week), body mass index (BMI, <18.5, 18.5–24.9, 25.0–29.9, 30.0–34.9, and ≥35.0 kg/m^2^), healthy eating index, of which higher scores represent better overall diet quality and adherence to the dietary guidelines (ethnicity- and sex-specific quintiles), history of diabetes, hypertension, dyslipidemia, CHD, and stroke (yes and no), and hormone replacement therapy (yes and no; for postmenopausal women only). For SWHS and SMHS participants, we further adjusted for refined carbohydrate intake (sex-specific quintiles) as a potential confounder because of strong positive associations between refined carbohydrate intake and cardiometabolic diseases in the SWHS and SMHS [28,29]. As missing rates of covariates were mostly less than 1%, missing values were assigned cohort- and sex-specific median (continuous variables) or mode (categorical variables) values of the non-missing covariates.

### 2.5. Statistical Analyses

Correlations between dietary cholesterol intake and other major nutrients and food items were assessed using generalized linear models adjusted for age, sex, and total energy intake separately by ethnic group. After adjusting for all covariates, we estimated the hazard ratios (HRs) and 95% confidence intervals (CIs) for mortality outcomes associated with dietary intakes of cholesterol and eggs using Cox proportional hazard regression models with restricted cubic splines. The follow-up time was computed from one year after the date of enrollment to the date of the last follow-up (i.e., death, end of follow-up, or loss to follow-up, whichever occurred earliest) and was used as the time scale. Goodness-of-fit tests using Schoenfeld residuals confirmed that the proportionality assumption was not violated. HRs (95% CIs) were estimated with 150 mg/day of cholesterol (half of the previously recommended limit) and 1 egg/week set as the references. In the models with restricted cubic splines, we included only participants within the 99th percentile of cholesterol/egg intake to reduce the potential effect of extreme values on the association.

Stratified analyses were performed by sex (men and women), age (<50 or ≥50, by the median age), BMI (<25 or ≥25 kg/m^2^), and history of diabetes, hypertension, dyslipidemia, CHD, and stroke (yes and no). Interactions (effect modifications) were assessed using the likelihood-ratio test for the cross-product term of dietary cholesterol/egg intake and the stratified variable.

A series of sensitivity analyses were conducted. For the first two sensitivity analyses, we excluded data in the first two years of follow-up or participants with a history of CVD (CHD or stroke). In other sensitivity analyses, we adopted a sequential adjustment approach including (1) age, sex, education, annual income, marital status, and total energy intake; (2) plus lifestyle (smoking status, smoking pack-years, alcohol consumption, and physical activity level) and hormone replacement therapy (women only); and (3) all covariates aforementioned and individual nutrients (i.e., saturated fat, polyunsaturated fat, carbohydrates, protein, fiber, sodium, and the combination of all as continuous variables) or cholesterol-containing foods (i.e., eggs, red meat, poultry, fish, shellfish, and total dairy products as continuous variables).

All statistical analyses were performed using SAS Enterprise Guide 7.1 (SAS Institute Inc., Cary, NC, USA). All *P* values were 2-sided and considered significant if *P* < 0.05.

## 3. Results

### 3.1. Characteristics of Study Participants

Of 202,429 participants (Table 1), the median (interquartile range) intake of cholesterol was 321.1 (197.7–511.5) mg/day in Black Americans, which was higher than 248.5 (162.0–389.4) in White Americans and 290.9 (188.2–399.8) in Chinese. Consistently, the intake of non-egg cholesterol was highest in Black Americans (median, 219.3 mg/day), but comparable between White Americans (175.8 mg/day) and Chinese (177.4 mg/day). The median (interquartile range) egg intake per week was 3.4 (0.8–6.9) in Black Americans, 1.5 (0.9–4.5) in White Americans, and 4.0 (2.0–7.0) in Chinese. The participants showed diverse sociodemographic characteristics and cardiometabolic risk factors. Generally, in the SCCS, White participants were more likely to have a college degree and high household income than Black participants. The SWHS/SMHS participants had a higher marriage percentage than the SCCS participants, but lower percentages of current smoking, heavy drinking, and history of chronic conditions such as diabetes, hypertension, dyslipidemia, CHD, and stroke. During the median follow-ups of 12.0 years in the SCCS and 15.5 years in the SWHS/SMHS, we ascertained a total of 30,899 deaths (12,113 deaths from cardiometabolic disease overall, 10,381 from CVD, and 1732 from diabetes) that included 9849 deaths (3917 deaths from cardiometabolic disease) in Black Americans, 4347 deaths (1432 deaths from cardiometabolic disease) in White Americans, and 16,703 deaths (6764 deaths from cardiometabolic disease) in Chinese. The rates of all-cause and cardiometabolic mortality were lower in Chinese than Black and White Americans, with the exception of stroke mortality.

Correlation coefficients between cholesterol intake and selected nutrients and foods are reported in Table S1. Across three ethnic groups, strong positive correlations (*r* > 0.50) were observed between cholesterol intake and intakes of protein, total fat, saturated fat, sodium, and eggs. In addition, significant but moderate correlations (0.2 < *r* < 0.5) were found between cholesterol intake and polyunsaturated fat, red meat, poultry, and fish/shellfish. Among Chinese, a strong inverse correlation existed between intakes of cholesterol and carbohydrate (*r* = −0.655).

### 3.2. Association between Dietary Cholesterol Intake and Mortality

**Figure 1:**
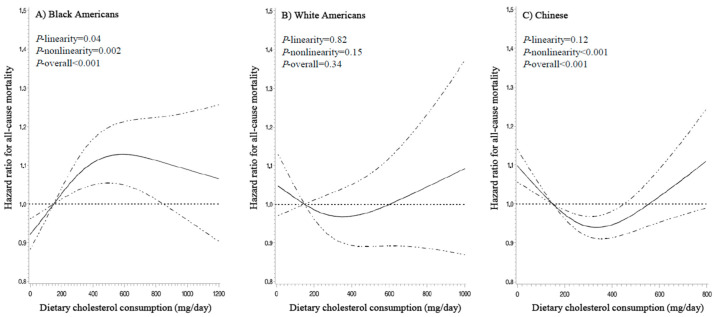
Dose-response relationships of dietary cholesterol intake with all-cause mortality. HRs (solid line) and 95% CIs (dashed line) were adjusted for age, sex, education, annual income, marital status, total energy intake, smoking status, smoking pack-years, alcohol consumption, physical activity level, body mass index, healthy eating index, history of diabetes, hypertension, dyslipidemia, coronary heart disease, and stroke, and hormone replacement therapy (for women only). Intake of refined carbohydrate was further adjusted for in the Chinese population. To minimize the potential effects of extreme values, participants with the top 1% of cholesterol intake were excluded from the analysis; 150 mg/day was set as the reference, and three knot positions were fitted at the 5th, 50th, and 95th percentiles. *P*-nonlinearity values for all-cause mortality were 0.002 in Blacks, 0.15 in Whites, and <0.001 in Chinese, respectively. Abbreviations: CI, confidence interval; HR, hazard ratio.

**Figure 2:**
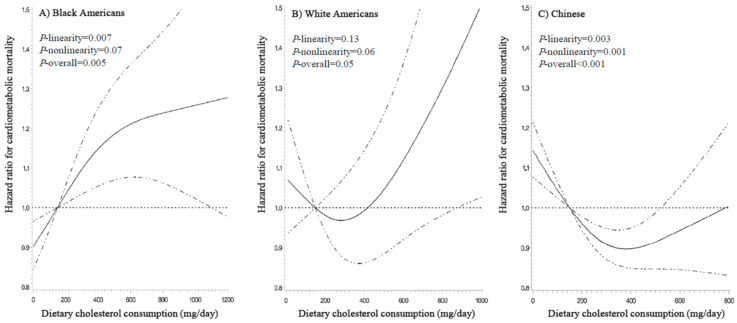
Dose-response relationships of dietary cholesterol intake with cardiometabolic mortality. HRs (solid line) and 95% CIs (dashed line) were adjusted for age, sex, education, annual income, marital status, total energy intake, smoking status, smoking pack-years, alcohol consumption, physical activity level, body mass index, healthy eating index, history of diabetes, hypertension, dyslipidemia, coronary heart disease, and stroke, and hormone replacement therapy (for women only). Intake of refined carbohydrate was further adjusted for in the Chinese population. To minimize the potential effects of extreme values, participants with the top 1% of cholesterol intake were excluded from the analysis; 150 mg/day was set as the reference, and three knot positions were fitted at the 5th, 50th, and 95th percentiles. *P*-nonlinearity for cardiometabolic mortality were 0.07, 0.06, and 0.001, respectively. Abbreviations: CI, confidence interval; HR, hazard ratio.

The associations of dietary cholesterol intake with mortality outcomes differed substantially across the three ethnic groups (Table 2 and Figure 1 and Figure 2). After adjusting for potential confounders, cholesterol intake showed a positive nonlinear association with all-cause mortality (*P*-linearity = 0.04, *P*-nonlinearity = 0.002, and *P*-overall < 0.001) and a positive linear association with cardiometabolic mortality among Black Americans (*P*-linearity = 0.007, *P*-nonlinearity = 0.07, and *P*-overall = 0.005): HRs (95% CIs) at 300, 450, 600, and 750 mg/day vs. 150 mg/day ranged from 1.07 (1.03–1.11) to 1.13 (1.05–1.21) for all-cause mortality and from 1.10 (1.03–1.16) to 1.23 (1.07–1.42) for cardiometabolic mortality. Of note, cholesterol intake showed a seemingly strong dose-response association with diabetes mortality in Black Americans (HR, 1.47; 95% CI, 1.01–2.14 for 750 mg/day vs. 150 mg/day; *P*-linearity = 0.05, *P*-nonlinearity = 0.10, and *P*-overall = 0.04; Table 2 and Figure S2).

Among White Americans, cholesterol intake was not significantly associated with either all-cause or cardiometabolic mortality (*P*-linearity ≥ 0.13, *P*-nonlinearity ≥ 0.06, and *P*-overall ≥ 0.05 for both; Table 2 and Figure 1 and Figure 2). The association with CHD mortality appeared to be linear: HRs (95% CI) were 1.39 (1.03–1.89) and 1.59 (1.07–2.36) for 600 and 750 mg/day vs. 150 mg/day (*P*-linearity = 0.02, *P*-nonlinearity = 0.74, and *P*-overall = 0.08; Table 2 and Figure S2).

On the contrary, cholesterol intake showed nonlinear inverse associations with all-cause (*P*-linearity = 0.12, *P*-nonlinearity < 0.001, and *P*-overall < 0.001) and cardiometabolic mortality (*P*-linearity = 0.003, *P*-nonlinearity = 0.001, and *P*-overall < 0.001) among Chinese (Table 2 and Figure 1 and Figure 2). HRs (95% CIs) at 300 and 450 mg/day vs. 150 mg/day was 0.94 (0.92–0.97) and 0.96 (0.92–1.00) for all-cause mortality and 0.91 (0.87–0.95) and 0.90 (0.85–0.96) for cardiometabolic mortality. However, the inverse associations seemed to disappear with cholesterol intake >500 mg/day. In addition, we found a strong inverse association with stroke mortality in Chinese (*P*-linearity < 0.001, *P*-nonlinearity = 0.01, and *P*-overall < 0.001; Table 2 and Figure S2): HRs (95% CIs) at 300 and 450 mg/day vs. 150 mg/day was 0.85 (0.80–0.91) and 0.81 (0.73–0.89).

The associations of cholesterol intake with all-cause mortality among Black Americans seemed to be modified by sex, age, BMI, diabetes, and hypertension (*P*-interaction ≤ 0.04 for all; Figure S3). The positive associations were more apparent in women, participants aged ≥ 50 years, and those with BMI ≥ 25 kg/m^2^, diabetes, or hypertension. In addition, a stronger association of cholesterol with cardiometabolic mortality was observed for Black Americans with hypertension (*P*-interaction = 0.03; Figure S4). There might be a positive association between dietary cholesterol with all-cause mortality among White men but not White women (*P* for interaction = 0.03; Figure S3). No other effect modifications were noted among White Americans or Chinese.

### 3.3. Association between Egg Intake and Mortality

While generally not significant or weak, the associations between egg intake and all-cause and cardiometabolic mortality also varied across the three ethnic groups (Table 3 and Figures S5 and S6). Compared with 1 egg/week, intakes of ≥7 eggs/week were associated with a 7% (HR, 1.07; 95% CI, 1.02–1.12) increase in all-cause mortality among Black Americans (*P*-linearity = 0.02, *P*-nonlinearity = 0.05, and *P*-overall = 0.01). Egg intake was not associated with cardiometabolic mortality in Black Americans (*P*-linearity = 0.07, *P*-nonlinearity = 0.88, and *P*-overall = 0.18). Among White Americans, egg intake was not associated with all-cause mortality (*P*-linearity = 0.23, *P*-nonlinearity = 0.88, and *P*-overall = 0.48) but was associated with 14% (HR, 1.14; 95% CI, 1.00–1.30) higher cardiometabolic mortality when intake was ≥10 eggs/week (*P*-linearity = 0.07, *P*-nonlinearity = 0.05, and *P*-overall = 0.03). Among Chinese, moderate intakes of 3–5 eggs/week were associated with ~5% lower all-cause mortality, while 10 eggs/week were associated with 6% (HR, 1.06; 95% CI, 1.01–1.11) higher all-cause mortality (*P*-linearity = 0.09, *P*-nonlinearity < 0.001, and *P*-overall < 0.001). Similarly, 5% lower cardiometabolic mortality was noted for 3–5 eggs/week vs. 1/week (*P*-linearity = 0.78, *P*-nonlinearity = 0.008, and *P*-overall = 0.03). There was no evidence for associations with mortality from major cardiometabolic subtypes in Chinese (*P*-linearity ≥ 0.08, *P*-nonlinearity ≥ 0.15, and *P*-overall ≥ 0.09), despite seemingly 9% (HR, 0.91; 95% CI, 0.84–0.99) lower stroke mortality for 5–7 eggs/week vs. 1 egg/week.

### 3.4. Sensitivity Analyses

Excluding data within the first two years of follow-up (Table S2) or participants with baseline CVD (Table S3) did not appreciably change the results across three ethnic groups. Sequential adjustments of sociodemographics, lifestyle and behavior factors, and preexisting medical conditions in three different models showed generally consistent results (Model 1–3; Tables S4 and S5). The results also remained stable after additional adjustments for nutrients such as saturated fat and sodium or cholesterol-containing foods (Model 3+nutrients/foods; Tables S4 and S5).

## 4. Discussion

In this study of 68,149 low-income Black and White Americans in the southeastern US and 134,280 Chinese in Shanghai, China, we documented possible ethnicity-specific associations of dietary cholesterol and eggs with all-cause and cardiometabolic mortality. Overall, cholesterol intake showed positive associations with all-cause and cardiometabolic mortality in Black Americans, null associations in White Americans, and nonlinear inverse associations in Chinese. Similar but modest associations were observed for egg intake. The ethnicity-specific patterns observed in the cholesterol/eggs-mortality associations remained robust in most stratified analyses by major CVD risk factors or preexisting conditions and a series of sensitivity analyses.

The epidemiologic evidence regarding dietary cholesterol/eggs with health outcomes among non-White populations has been limited. To our knowledge, only two studies have examined the associations of cholesterol intake with cardiometabolic disease or all-cause mortality among Black Americans, including a subgroup analysis of 9204 Black Americans from a pooling project of six cohorts in the US and an analysis of 7683 Black Americans using the NHANES 1999–2014 data [12,30]. Consistent with our findings, both studies reported positive associations of cholesterol intake with all-cause mortality among Black Americans. However, neither study, with small sample sizes, reported varied cholesterol–mortality associations across ethnicities [12,30]. Among White Americans, the null association between cholesterol and cardiometabolic mortality we observed was in line with the findings from two systematic reviews of 17 cohort studies [6,10], which included primarily white populations. Among Chinese, we found nonlinear inverse associations with all-cause and cardiometabolic mortality for cholesterol intake up to 450 mg/day in Chinese; however, the China Health and Nutrition Survey (CHNS) study among 18,914 participants found no association between total cholesterol and overall mortality, despite a potential inverse association for cholesterol from eggs [31]. The discrepancies may be due to different dietary patterns and cardiometabolic risk profiles in two studies: our Chinese participants were middle-aged residents (40 years or above) from affluent urban Shanghai, while the CHNS participants were younger (20 years or above) from across China.

The pool of cholesterol in the body is determined by the balance among de novo synthesis, dietary intake, absorption, metabolism, enterohepatic recirculation, and excretion [32]. The divergent associations of dietary cholesterol with cardiometabolic mortality may be linked to different cholesterol intake levels and food sources across ethnicities. In the current study, intakes of overall cholesterol and non-egg cholesterol were higher in Black Americans than in White Americans and Chinese, as consistently found in national surveys in the US (320 mg/day in non-Hispanic Blacks and 282 mg/day in non-Hispanic Whites in 2013–2014) [33] and China (266 mg/day in 2011) [34]. Some non-egg cholesterol sources, such as red and processed meat, are also sources of saturated fat and sodium, which may confound the association between cholesterol intake and cardiometabolic risk [35]. However, our results remained consistent after further controlling for saturated fat intake and other nutrients such as carbohydrates and sodium or major cholesterol-containing foods. Still, we cannot rule out the possibility of residual confounding due to imperfect dietary measures. Nevertheless, dietary cholesterol intake only slightly increases serum cholesterol levels, as supported by findings from a meta-analysis of 17 trials that 100 mg/day cholesterol intake only increased total cholesterol by 0.056 mmol/L and low-density lipoprotein cholesterol by 0.050 mmol/L [36]. Besides dietary intake, it is also uncertain whether cholesterol absorption and de novo synthesis, which are largely genetically determined [32], are different across ethnicities; future feeding studies including multi-ethnic participants are needed. In addition, our Black and White participants were mostly from low-income communities, with the majority having an annual household income ≤ 15,000 USD. The risk profile, e.g., low socioeconomic status, unhealthy lifestyles (e.g., current smoking and heavy drinking), and underlying metabolic risk factors (e.g., hypertension and diabetes), in this disadvantaged American population were vastly different from those in urban Chinese, which may confound and modify the associations between dietary cholesterol and mortality [6]. It is likely that the different risk profiles across ethnicities may partly account for the differential associations observed in our study.

To date, several meta-analyses have documented a lack of associations between egg consumption and cardiovascular events or all-cause mortality [7,8,9,11]. In a recent analysis of three international prospective studies, including ∼177,000 individuals from 50 countries, a higher intake of eggs (≥7 eggs/week vs. < 1 egg/week) was also not associated with blood lipids, major CVD events, or all-cause mortality [37]. However, none of these studies examined the link between egg consumption and cardiometabolic health across ethnicities. Our study, for the first time, showed contrasting results for mortality outcomes associated with egg intake among Black and White Americans and Chinese. In our largest ever sample of Black participants on this topic, we noted significant positive associations between egg consumption and all-cause mortality, which was in agreement with the finding from the subgroup analysis in the previously mentioned pooling study of six US cohorts [12]. However, the same pooling study also demonstrated higher risks associated with egg consumption in other US racial groups. In our analyses among White Americans, egg intake was generally not associated with all-cause or cardiometabolic mortality, although there may be an increased risk when the intake was ≥10 eggs/week, which was rare. Our finding in the White Americans was consistent with the recent report from the Nurses’ Health Study and Health Professionals’ Follow-Up Study that consumption of 1 egg per day was not associated with incident CVD [11]. In our analyses among the Chinese population, a moderate intake of eggs up to 5 vs. 1 egg/week was associated with ~5% reduced risk for both all-cause and cardiometabolic mortality; however, a high intake of 10 eggs/week was associated with 6% increased all-cause mortality. Three community-based cohorts in China have examined this topic so far: while the Guangzhou Biobank Cohort Study (28,024 participants) did not find any associations for all-cause and CVD mortality [9], the multi-site China Kadoorie Biobank study (461,213 participants) and the CHNS study (18,914 participants) showed dose-response inverse relations of egg intake with all-cause mortality [31] or CVD risk [38]. Our findings were largely consistent with findings in the latter two studies, although there remains a concern for increased all-cause mortality for consuming 10 or more eggs/week among Chinese. Across populations, a 2020 meta-analysis of 28 prospective studies including 1.72 million participants showed that 1 egg/day was not associated with a risk of CVD and its subtypes, whereas with stratification by geographic regions, a significant inverse association (8% decrease in CVD risk for 1 egg/day increase) was observed in Asian cohorts but not in US or European cohorts, which supports our ethnicity-specific findings [11]. Besides the different intake levels of eggs (higher in Chinese and Black Americans than in White Americans) in our study, the possible heterogeneity across ethnicities may reflect different egg consumption patterns: eggs were typically consumed with meats and refined grains in western populations but with vegetables in Chinese populations [30]. Thus, caution should be taken in interpreting the harms or benefits of eggs across ethnicities due to the observational nature of the current analyses, as there remains possible residual confounding from sociodemographics and lifestyle factors.

In our subgroup analyses, the positive cholesterol–mortality associations were stronger among Black participants with diabetes and hypertension. Two separate meta-analyses have consistently shown that higher egg consumption could be associated with a higher risk of CVD among patients with diabetes [7,8], which supports our findings to some extent. Since patients with diabetes can have a high risk of dyslipidemia [39] and elevated cholesterol synthesis [40], it is postulated that cholesterol intake might heighten the risk of CVD associated with diabetes and hypertension. Of note, the most recent scientific advice from the American Heart Association recommends that particular caution should be taken with high cholesterol intake in individuals with cardiovascular risk factors such as dyslipidemia, diabetes, and hypertension [6]. Future studies are needed to understand the underlying mechanisms for the elevated CVD or all-cause mortality associated with high cholesterol intake in people with CVD risk factors.

The major strengths of our study include a large sample size of ethnically diverse populations, the prospective nature, and well-characterized study participants. However, certain limitations should be recognized in the interpretation and extrapolation of our findings. First, measurement errors were inherent in dietary assessments via FFQs and may differ between US and Chinese cohorts. However, all the FFQs were designed to capture commonly consumed foods in respective populations and had been validated against food recalls. Moreover, the measurement errors were likely non-differential among participants within each cohort with or without mortality outcomes of interest. Second, cholesterol and egg intakes were estimated only at the baseline, and their changes over time could not be quantified in the current analyses. Since participants with CVD risk factors may be more likely to reduce their intake of cholesterol or eggs, the magnitude could be underestimated for positive associations or overestimated for inverse associations. Third, although we comprehensively adjusted for confounding factors and conducted a series of subgroup and sensitivity analyses, residual confounding cannot be ruled out, as some potential confounders could not be precisely ascertained, such as the ways of cooking eggs. Fourth, the White and Black Americans in our study were socioeconomically disadvantaged, and the Chinese participants were residents from urban Shanghai, so our present findings may not be readily generalizable to other populations of similar ethnicities, and caution should be taken in interpreting the distinct associations across ethnicities with different background diets and cardiometabolic profiles.

## 5. Conclusions

In conclusion, our findings suggest ethnicity-specific associations of dietary cholesterol and egg intakes with mortality outcomes. We observed positive associations of dietary cholesterol intake with all-cause and cardiometabolic mortality among low-income Black Americans but null associations among White Americans. In contrast, inverse associations of dietary cholesterol intake with mortality were observed among Chinese adults when the intake was as moderate as up to 500 mg/day. Similar ethnicity-specific associations with mortality outcomes were observed for egg intakes. Owing to potential residual confounding and different cardiometabolic risk profiles, our findings need to be validated in future prospective studies across ethnicities. Future studies are also needed to examine dietary cholesterol and eggs with incident CVD and other diseases among diverse populations.

## Figures and Tables

**Table 1:** Characteristics of study participants ^1^.

	Blacks, *n* = 47,789	Whites, *n* = 20,360	Chinese, *n* = 134,280
Age, years	50.0 (45.0–56.0)	52.0 (46.0–59.0)	51.0 (45.0–61.0)
Men, %	41.5	39.9	45.5
Educational attainment, %			
<High school graduation	31.1	23.3	50.2
High school graduation	34.0	32.0	31.6
Some college	24.9	25.9	10.4
≥University degree	10.0	18.8	7.8
Cohort-specific income level ^2^, %			
Low	59.6	47.4	14.4
Lower-middle	22.4	18.5	40.3
Upper-middle	12.7	16.5	31.3
High	5.4	17.6	14.0
Married, %	29.4	49.2	92.8
Smoking status, %			
Never smoker	37.4	33.5	66.8
Former smoker	19.9	27.7	5.2
Current smoker	42.7	38.8	28.0
Smoking pack-years, among ever-smokers	14.1 (6.8–25.0)	26.3 (13.0–42.3)	21.6 (12.3–31.5)
Alcohol consumption ^3^, %			
None	45.2	49.2	83.9
Moderate drinking	35.4	38.0	9.9
Heavy drinking	19.4	12.8	6.2
Total physical activity, MET-hours/week	124.8 (64.4–218.4)	120.4 (63.0–208.2)	78.9 (51.1–111.7)
Body mass index, kg/m^2^	29.3 (25.1–34.7)	28.7 (24.7–33.8)	23.7 (21.6–25.9)
Menopause, among women, %	64.1	74.3	49.2
HRT, among women, %	7.8	13.9	2.1
Medical conditions at baseline, %			
Diabetes	22.0	19.2	5.2
Hypertension	57.4	48.2	27.1
Dyslipidemia	29.5	40.8	1.9
Coronary heart disease	5.6	9.2	6.4
Stroke	6.0	6.6	2.3
Dietary factors			
Total energy, kcal/day	2301 (1566–3457)	1988 (1435–2800)	1728 (1469–2038)
Dietary cholesterol intake, mg/day	321.1 (197.7–511.5)	248.5 (162.0–389.4)	290.9 (188.2–399.8)
Cholesterol from eggs	91.5 (21.4–183.1)	38.6 (22.5–118.5)	106.3 (53.1–186.0)
Cholesterol from non-egg sources	219.3 (140.2–346.6)	175.8 (118.8–266.4)	177.4 (123.8–241.4)
No. of eggs per week	3.4 (0.8–6.9)	1.5 (0.9–4.5)	4.0 (2.0–7.0)
Healthy Eating Index	57.4 (49.4–65.7)	56.8 (48.0–66.2)	34.0 (30.7–36.7)
Outcomes			
Follow-up, years	12.0 (10.0–14.0)	12.0 (9.0–13.0)	15.5 (12.7–18.8)
Deaths, *n* (per 100,000 person years)	9849 (1782.0)	4347 (1977.4)	16,703 (805.9)
Cardiometabolic disease	3917 (708.7)	1432 (651.4)	6764 (326.4)
Cardiovascular disease	3282 (593.8)	1253 (570.0)	5846 (282.1)
Coronary heart disease	1179 (213.3)	595 (270.7)	2182 (105.3)
Stroke	557 (100.8)	142 (64.6)	2955 (142.6)
Diabetes	635 (114.9)	179 (81.4)	918 (44.3)

Abbreviations: HRT, hormone replacement therapy; n, Number; MET, metabolic equivalent of task. ^1^ Data are presented as median (interquartile range) of continuous variables or proportion (%) of categorical variables (except mortality outcomes). ^2^ Annual income was defined as low, lower-middle, upper-middle, and high (for the SCCS: annual household income < 15,000 US dollars (USD), ≥15,000 to <25,000 USD, ≥25,000 to <50,000 USD, and ≥50,000 USD; for the SWHS: annual household income < 10,000 CNY, ≥10,000 to <20,000 CNY, ≥20,000 to < 30,000 CNY, and ≥30,000 CNY; and for the SMHS: annual personal income < 6000 Chinese Yuan (CNY), ≥6000 to <12,000 CNY, ≥12,000 to <4000 CNY, and ≥24,000 CNY). ^3^ Heavy drinking was defined as alcohol consumption of > 2 drinks per day in men or alcohol consumption of >1 drink per day in women; moderate drinking was defined as alcohol consumption of >0 to ≤2 drinks per day in men or >0 to ≤1 drink per day in women.

**Table 2:** All-cause and cause-specific mortality in relation to dietary cholesterol intake.

	Blacks, *n* = 47,789	Whites, *n* = 20,360	Chinese, *n* = 134,280
	HR (95% CI) ^1^	HR (95% CI) ^1^	HR (95% CI) ^1,2^
**All causes**			
150 mg/day	1 (reference)	1 (reference)	1 (reference)
300 mg/day	1.07 (1.03–1.11)	0.97 (0.91–1.03)	0.94 (0.92–0.97)
450 mg/day	1.12 (1.05–1.19)	0.97 (0.89–1.06)	0.96 (0.92–1.00)
600 mg/day	1.13 (1.05–1.21)	1.00 (0.89–1.12)	1.02 (0.95–1.09)
750 mg/day	1.12 (1.02–1.22)	1.03 (0.89–1.20)	1.09 (0.98–1.20)
*P*-linearity ^3^	0.04	0.82	0.12
*P*-nonlinearity ^4^	0.002	0.15	<0.001
*P*-overall ^5^	<0.001	0.34	<0.001
**Cardiometabolic disease**			
150 mg/day	1 (reference)	1 (reference)	1 (reference)
300 mg/day	1.10 (1.03–1.16)	0.97 (0.87–1.08)	0.91 (0.87–0.95)
450 mg/day	1.17 (1.06–1.28)	1.02 (0.87–1.19)	0.90 (0.85–0.96)
600 mg/day	1.21 (1.08–1.36)	1.12 (0.92–1.36)	0.94 (0.85–1.05)
750 mg/day	1.23 (1.07–1.42)	1.25 (0.97–1.62)	0.99 (0.84–1.17)
*P*-linearity ^3^	0.007	0.13	0.003
*P*-nonlinearity ^4^	0.07	0.06	0.001
*P*-overall ^5^	0.005	0.05	<0.001
**Coronary heart disease**			
150 mg/day	1 (reference)	1 (reference)	1 (reference)
300 mg/day	1.03 (0.92–1.14)	1.10 (0.93–1.29)	0.96 (0.89–1.04)
450 mg/day	1.05 (0.88–1.24)	1.23 (0.96–1.56)	0.98 (0.87–1.10)
600 mg/day	1.06 (0.85–1.30)	1.39 (1.03–1.89)	1.02 (0.85–1.24)
750 mg/day	1.06 (0.82–1.37)	1.59 (1.07–2.36)	1.07 (0.80–1.44)
*P*-linearity ^3^	0.69	0.02	0.75
*P*-nonlinearity ^4^	0.73	0.74	0.26
*P*-overall ^5^	0.87	0.08	0.51
**Stroke**			
150 mg/day	1 (reference)	1 (reference)	1 (reference)
300 mg/day	1.09 (0.94–1.28)	0.91 (0.65–1.26)	0.85 (0.80–0.91)
450 mg/day	1.17 (0.91–1.50)	0.89 (0.54–1.45)	0.81 (0.73–0.89)
600 mg/day	1.23 (0.90–1.67)	0.91 (0.46–1.77)	0.81 (0.68–0.97)
750 mg/day	1.27 (0.88–1.84)	0.94 (0.37–2.36)	0.82 (0.63–1.07)
*P*-linearity ^3^	0.23	0.78	<0.001
*P*-nonlinearity ^4^	0.42	0.84	<0.001
*P*-overall ^5^	0.59	0.61	0.01
**Diabetes**			
150 mg/day	1 (reference)	1 (reference)	1 (reference)
300 mg/day	1.21 (1.04–1.40)	0.88 (0.65–1.19)	1.00 (0.89–1.12)
450 mg/day	1.36 (1.07–1.73)	0.81 (0.51–1.28)	1.04 (0.87–1.23)
600 mg/day	1.44 (1.07–1.95)	0.77 (0.42–1.41)	1.10 (0.83–1.45)
750 mg/day	1.47 (1.01–2.14)	0.74 (0.33–1.66)	1.16 (0.76–1.78)
*P*-linearity ^3^	0.05	0.42	0.65
*P*-nonlinearity ^4^	0.10	0.70	0.60
*P*-overall ^5^	0.04	0.67	0.79

Abbreviations: CI, confidence interval; HR, hazard ratio. ^1^ Adjusted for age, sex, education, annual income, marital status, total energy intake, smoking status, smoking pack-years, alcohol consumption, physical activity level, body mass index, healthy eating index, history of diabetes, hypertension, dyslipidemia, coronary heart disease, and stroke, and hormone replacement therapy (for women only). ^2^ Intake of refined carbohydrate was further adjusted for in the Chinese population. ^3^ *P*-linearity values were obtained from Cox proportional hazard regression models with the exposure modeled as a linear term. ^4^ *P*-nonlinearity values were obtained from Cox proportional hazard regression models with the exposure modeled as both cubic spline and linear terms. ^5^ *P*-overall values were obtained for Cox proportional hazard regression models with the exposure modeled as a cubic spline term.

**Table 3:** All-cause and cause-specific mortality in relation to egg intake.

	Blacks, *n* = 47,789	Whites, *n* = 20,360	Chinese, *n* = 134,280
	HR (95% CI) ^1^	HR (95% CI) ^1^	HR (95% CI) ^1,2^
**All causes**			
1 egg/week	1 (reference)	1 (reference)	1 (reference)
3 eggs/week	1.04 (1.01–1.06)	1.01 (0.97–1.05)	0.95 (0.93–0.98)
5 eggs/week	1.06 (1.02–1.10)	1.02 (0.96–1.08)	0.96 (0.92–0.99)
7 eggs/week	1.07 (1.02–1.12)	1.03 (0.97–1.10)	0.99 (0.96–1.03)
10 eggs/week	1.07 (1.02–1.12)	1.05 (0.97–1.14)	1.06 (1.01–1.11)
*P*-linearity ^3^	0.02	0.23	0.09
*P*-nonlinearity ^4^	0.05	0.88	<0.001
*P*-overall ^5^	0.01	0.48	<0.001
**Cardiometabolic disease**			
1 egg/week	1 (reference)	1 (reference)	1 (reference)
3 eggs/week	1.01 (0.97–1.05)	0.96 (0.89–1.04)	0.95 (0.91–0.99)
5 eggs/week	1.03 (0.96–1.09)	0.97 (0.87–1.08)	0.95 (0.90–1.00)
7 eggs/week	1.04 (0.97–1.12)	1.02 (0.91–1.14)	0.98 (0.92–1.03)
10 eggs/week	1.07 (0.99–1.15)	1.14 (1.00–1.30)	1.03 (0.95–1.11)
*P*-linearity ^3^	0.07	0.07	0.78
*P*-nonlinearity*^4^*	0.88	0.05	0.008
*P*-overall ^5^	0.18	0.03	0.03
**Coronary heart disease**			
1 egg/week	1 (reference)	1 (reference)	1 (reference)
3 eggs/week	1.05 (0.97–1.13)	1.00 (0.89–1.12)	0.96 (0.90–1.04)
5 eggs/week	1.07 (0.95–1.20)	1.03 (0.87–1.21)	0.97 (0.88–1.07)
7 eggs/week	1.06 (0.93–1.21)	1.08 (0.91–1.28)	1.01 (0.92–1.12)
10 eggs/week	1.01 (0.88–1.16)	1.20 (0.98–1.47)	1.08 (0.95–1.24)
*P*-linearity ^3^	0.85	0.09	0.34
*P*-nonlinearity ^4^	0.16	0.44	0.15
*P*-overall ^5^	0.36	0.18	0.22
**Stroke**			
1 egg/week	1 (reference)	1 (reference)	1 (reference)
3 eggs/week	1.01 (0.90–1.12)	0.93 (0.73–1.19)	0.94 (0.88–1.00)
5 eggs/week	1.02 (0.86–1.20)	0.96 (0.69–1.34)	0.91 (0.84–0.99)
7 eggs/week	1.03 (0.85–1.24)	1.07 (0.75–1.51)	0.91 (0.84–0.99)
10 eggs/week	1.05 (0.86–1.28)	1.34 (0.89–2.02)	0.92 (0.81–1.03)
*P*-linearity ^3^	0.58	0.20	0.08
*P*-nonlinearity ^4^	0.92	0.25	0.18
*P*-overall ^5^	0.86	0.22	0.09
**Diabetes**			
1 egg/week	1 (reference)	1 (reference)	1 (reference)
3 eggs/week	1.05 (0.95–1.16)	0.93 (0.75–1.15)	0.99 (0.88–1.11)
5 eggs/week	1.10 (0.93–1.29)	0.91 (0.67–1.22)	1.02 (0.88–1.19)
7 eggs/week	1.13 (0.95–1.35)	0.91 (0.66–1.24)	1.09 (0.93–1.27)
10 eggs/week	1.18 (0.97–1.42)	0.93 (0.63–1.38)	1.20 (0.99–1.46)
*P*-linearity ^3^	0.10	0.68	0.08
*P*-nonlinearity ^4^	0.75	0.61	0.37
*P*-overall ^5^	0.24	0.81	0.14

Abbreviations: CI, confidence interval; HR, hazard ratio. ^1^ Adjusted for age, sex, education, annual income, marital status, total energy intake, smoking status, smoking pack-years, alcohol consumption, physical activity level, body mass index, healthy eating index, history of diabetes, hypertension, dyslipidemia, coronary heart disease, and stroke, and hormone replacement therapy (for women only). ^2^ Intake of refined carbohydrate was further adjusted for in the Chinese population. ^3^ *P*-linearity values were obtained from Cox proportional hazard regression models with the exposure modeled as a linear term. ^4^ *P*-nonlinearity values were obtained from Cox proportional hazard regression models with the exposure modeled as both cubic spline and linear terms. ^5^ *P*-overall values were obtained for Cox proportional hazard regression models with the exposure modeled as a cubic spline term.

## Data Availability

The data described in the manuscript will be made available upon reasonable request and approval of the corresponding author of the manuscript and the Data Use Committee of each included cohort.

## Supplementary Materials

The following are available online at https://www.mdpi.com/article/10.3390/nu13062094/s1. Table S1. Correlations between dietary cholesterol intake and selected nutrients and food items. Table S2. Total and cause-specific mortality in relation to dietary cholesterol and egg intakes: excluding data within the first two years of follow-up. Table S3. Total and cause-specific mortality in relation to dietary cholesterol and egg intakes: excluding participants with a history of cardiovascular disease. Table S4. All-cause and cardiometabolic mortality in relation to dietary cholesterol intake: sequential adjustments of covariates and dietary factors. Table S5. All-cause and cardiometabolic mortality in relation to egg intake: sequential adjustments of covariates and dietary factors. Figure S1. Flowchart of participant selection and exclusion in three cohorts. Figure S2. Dose–response relationships of dietary cholesterol intake with diabetes mortality among Blacks, CHD mortality among Whites, and stroke mortality among Chinese. Figure S3. All-cause mortality in relation to dietary cholesterol intake: subgroup analyses. Figure S4. Cardiometabolic mortality in relation to dietary cholesterol intake: subgroup analyses. Figure S5. Dose–response relationships of egg intake with all-cause mortality. Figure S6. Dose-response relationships of egg intake with cardiometabolic mortality.
