# Supplementary material for: Cholesterol and Egg Intakes with Cardiometabolic and All-Cause Mortality among Chinese and Low-Income Black and White Americans
Source: Nutrients. 2021 Jun 19;13(6):2094. doi: 10.3390/nu13062094 (PMC8234137; doi:10.3390/nu13062094)
Supplement: Supplementary file 1 [file nutrients-13-02094-s001.zip › nutrients-1229345-supplementary.pdf]

nutrients

MDPI

Supplementary

Cholesterol and Egg Intakes with Cardiometabolic and All-Cause Mortality among Chinese and Low-Income Black and White Americans

Xiong-Fei Pan [1,#], Jae Jeong Yang [1,#], Loren P. Lipworth [1], Xiao-Ou Shu [1], Hui Cai [1], Mark D. Steinwandel [2], William J. Blot [1,2], Wei Zheng [1] and Danxia Yu [1,*]

Table of contents

Page 3 Table S1. Correlations between dietary cholesterol intake and selected nutrients and food items

Page 4 Table S2. Total and cause-specific mortality in relation to dietary cholesterol and egg intakes: excluding data within the first two years of follow-up

Page 5 Table S3. Total and cause-specific mortality in relation to dietary cholesterol and egg intakes: excluding participants with a history of cardiovascular disease

Page 6 Table S4. All-cause and cardiometabolic mortality in relation to dietary cholesterol intake: sequential adjustments of covariates and dietary factors

Page 8 Table S5. All-cause and cardiometabolic mortality in relation to eggs intake: sequential adjustments of covariates and dietary factors

Page 10 Figure S1. Flowchart of participant selection and exclusion in three cohorts

Page 11 Figure S2. Dose-response relationships of dietary cholesterol intake with diabetes mortality among Blacks, CHD mortality among Whites, and stroke mortality among Chinese

HRs (solid line) and 95% CIs (dashed line) were adjusted for age, sex, education, annual income, marital status, total energy intake, smoking status, smoking pack-years, alcohol consumption, physical activity level, body mass index, healthy eating index, history of diabetes, hypertension, dyslipidemia, coronary heart disease, and stroke, and hormone replacement therapy (for women only). Intake of refined carbohydrate was further adjusted for in the Chinese population. To minimize potential effects of extreme values, participants with the top 1% of cholesterol intake were excluded from the analysis. 150 mg/day was set as the reference and three knots were fitted at the 5th, 50th, and 95th percentiles. P-nonlinearity values were 0.10 for diabetes mortality in Blacks, 0.74 for coronary heart disease mortality in Whites, and <0.001 for stroke mortality in Chinese. Abbreviations: CHD, coronary heart disease, CI, confidence interval; HR, hazard ratio.

Page 12 Figure S3. All-cause mortality in relation to dietary cholesterol intake: subgroup analyses

HRs (95% CIs) represent 600 mg/day vs. 150 mg/day of dietary cholesterol consumption. Estimates were adjusted for age, sex, education, annual income, marital status, total energy intake, smoking status, smoking pack-years, alcohol consumption, physical activity level, body mass index, healthy eating index, history of diabetes, hypertension, dyslipidemia, coronary heart disease, and stroke, and hormone replacement therapy (for women only). Intake of refined carbohydrate was further adjusted for in the Chinese population. Abbreviations: CI, confidence interval; HR, hazard ratio.

Page 13 Figure S4. Cardiometabolic mortality in relation to dietary cholesterol intake: subgroup analyses

HRs (95% CIs) represent 600 mg/day vs. 150 mg/day of dietary cholesterol consumption. Estimates were adjusted for age, sex, education, annual income, marital status, total energy intake, smoking status, smoking pack-years, alcohol consumption, physical activity level, body mass index, healthy eating index, history of diabetes, hypertension,

dyslipidemia, coronary heart disease, and stroke, and hormone replacement therapy (for women only). Intake of refined carbohydrate was further adjusted for in the Chinese population. Abbreviations: CI, confidence interval; HR, hazard ratio.

Page 14 Figure S5. Dose-response relationships of egg intake with all-cause mortality

HRs (solid line) and 95% CIs (dashed line) were adjusted for age, sex, education, annual income, marital status, total energy intake, smoking status, smoking pack-years, alcohol consumption, physical activity level, body mass index, healthy eating index, history of diabetes, hypertension, dyslipidemia, coronary heart disease, and stroke, and hormone replacement therapy (for women only). Intake of refined carbohydrate was further adjusted for in the Chinese population. To minimize potential effects of extreme values, participants with the top 1% of egg intake were excluded from the analysis. One egg consumed per week was set as the reference, and three knot positions were fitted at the 5th, 50th, and 95th percentiles. *P*-nonlinearity values for all-cause mortality were 0.05 in Blacks, 0.88 in Whites, and <0.001 in Chinese, respectively. Abbreviations: CI, confidence interval; HR, hazard ratio.

Page 15 Figure S6. Dose-response relationships of egg intake with cardiometabolic mortality

HRs (solid line) and 95% CIs (dashed line) were adjusted for age, sex, education, annual income, marital status, total energy intake, smoking status, smoking pack-years, alcohol consumption, physical activity level, body mass index, healthy eating index, history of diabetes, hypertension, dyslipidemia, coronary heart disease, and stroke, and hormone replacement therapy (for women only). Intake of refined carbohydrate was further adjusted for in the Chinese population. To minimize potential effects of extreme values, participants with the top 1% of egg intake were excluded from the analysis. One egg consumed per week was set as the reference, and three knot positions were fitted at the 5th, 50th, and 95th percentiles. *P*-nonlinearity values for cardiometabolic mortality were 0.88, 0.05, and 0.008, respectively. Abbreviations: CI, confidence interval; HR, hazard ratio.

Table S1. Correlations between dietary cholesterol intake and selected nutrients and food items [1].

	**Blacks (*n* = 47,789)**	**Whites (*n* = 20,356)**	**Chinese (*n* = 134,280)**
Eggs	0.666	0.759	0.866
Carbohydrate	−0.307	−0.283	−0.655
Protein	0.701	0.646	0.617
Total fat	0.568	0.575	0.589
Saturated fat	0.575	0.574	0.611
Polyunsaturated fat	0.356	0.343	0.382
Fiber	−0.056	−0.018	0.093
Sodium	0.581	0.576	0.565
Red meat	0.440	0.417	0.326
Poultry	0.318	0.264	0.303
Dairy	−0.001 [2]	−0.010 [2]	0.261
Fish	0.294	0.211	0.364
Shellfish	0.316	0.229	0.330
Soy products	−0.018	−0.033	0.083
Legumes	0.029	0.069	0.058
Vegetable	0.039	0.055	0.119
Fruits	−0.145	−0.095	0.103
Wheat products	0.054	0.055	0.024

[1] Partial correlation coefficients were estimated using the generalized linear model adjusted for age, sex, and total energy intake.

[2] Non-significant correlation.

Table S2. Total and cause-specific mortality in relation to dietary cholesterol and egg intakes: excluding data within the first two years of follow-up.

	Blacks (*n* = 47,278)	Whites (*n* = 20,109)	Chinese (*n* = 133,792)
	HR (95% CI) [1]	HR (95% CI) [1]	HR (95% CI) [1,2]
Dietary cholesterol			
**All causes**			
150 mg/day	1 (reference)	1 (reference)	1 (reference)
300 mg/day	1.08 (1.03–1.12)	0.97 (0.91–1.03)	0.94 (0.91–0.97)
450 mg/day	1.12 (1.05–1.19)	0.98 (0.89–1.07)	0.95 (0.92–0.99)
600 mg/day	1.13 (1.05–1.21)	1.01 (0.90–1.14)	1.01 (0.95–1.08)
750 mg/day	1.11 (1.02–1.22)	1.05 (0.90–1.23)	1.08 (0.97–1.20)
P-linearity [3]	0.05	0.67	0.08
P-nonlinearity [4]	0.001	0.13	< 0.001
P-overall [5]	< 0.001	0.28	< 0.001
**Cardiometabolic disease**			
150 mg/day	1 (reference)	1 (reference)	1 (reference)
300 mg/day	1.09 (1.03–1.16)	0.98 (0.88–1.09)	0.91 (0.87–0.95)
450 mg/day	1.16 (1.05–1.28)	1.02 (0.87–1.20)	0.89 (0.84-0.96)
600 mg/day	1.19 (1.06–1.35)	1.11 (0.91–1.36)	0.92 (0.83–1.03)
750 mg/day	1.21 (1.05–1.40)	1.23 (0.94–1.61)	0.96 (0.81–1.14)
P-linearity [3]	0.02	0.17	0.001
P-nonlinearity [4]	0.08	0.11	0.003
P-overall [5]	0.01	0.11	< 0.001
No. of eggs consumed per week			
**All causes**			
1 egg/week	1 (reference)	1 (reference)	1 (reference)
3 eggs/week	1.04 (1.01–1.06)	1.01 (0.96–1.05)	0.95 (0.93–0.98)
5 eggs/week	1.06 (1.02–1.10)	1.02 (0.96–1.08)	0.95 (0.92–0.99)
7 eggs/week	1.07 (1.02–1.12)	1.03 (0.97–1.10)	0.99 (0.95–1.02)
10 eggs/week	1.07 (1.01–1.12)	1.06 (0.98–1.15)	1.05 (1.00–1.10)
P-linearity [3]	0.03	0.16	0.14
P-nonlinearity [4]	0.06	0.76	< 0.001
P-overall [5]	0.02	0.36	< 0.001
**Cardiometabolic disease**			
1 egg/week	1 (reference)	1 (reference)	1 (reference)
3 eggs/week	1.01 (0.97–1.05)	0.96 (0.89–1.04)	0.95 (0.91–0.99)
5 eggs/week	1.02 (0.95–1.09)	0.97 (0.87–1.09)	0.95 (0.90–1.00)
7 eggs/week	1.03 (0.96–1.11)	1.02 (0.91–1.14)	0.97 (0.92–1.03)
10 eggs/week	1.06 (0.98–1.14)	1.13 (0.98–1.29)	1.02 (0.95–1.11)
P-linearity [3]	0.14	0.10	0.87
P-nonlinearity [4]	0.81	0.09	0.01
P-overall [5]	0.33	0.06	0.04

Abbreviations: CI, confidence interval; HR, hazard ratio.

[1] Adjusted for age, sex, education, annual income, marital status, total energy intake, smoking status, smoking pack-years, alcohol consumption, physical activity level, body mass index, healthy eating index, history of diabetes, hypertension, dyslipidemia, coronary heart disease, and stroke, and hormone replacement therapy (for women only).

[2] Intake of refined carbohydrate was further adjusted for in the Chinese population.

[3] *P*-linearity values were obtained from Cox proportional hazard regression models with the exposure modeled as a linear term.

[4] *P*-nonlinearity values were obtained from Cox proportional hazard regression models with the exposure modeled as both cubic spline and linear terms.

[5] *P*-overall values were obtained for Cox proportional hazard regression models with the exposure modeled as a cubic spline term

Table S3. Total and cause-specific mortality in relation to dietary cholesterol and egg intakes: excluding participants with a history of cardiovascular disease.

	Blacks (*n* = 42,825)	Whites (*n* = 17,548)	Chinese (*n* = 123,261)

	**HR (95% CI)** [1]	**HR (95% CI)** [1]	**HR (95% CI)** [1,2]
	Dietary cholesterol		
**All causes**			
150 mg/day	1 (reference)	1 (reference)	1 (reference)
300 mg/day	1.07 (1.02–1.11)	0.96 (0.90–1.03)	0.94 (0.91–0.97)
450 mg/day	1.10 (1.03–1.18)	0.96 (0.87–1.07)	0.95 (0.90–0.99)
600 mg/day	1.11 (1.02–1.20)	0.99 (0.87–1.13)	0.99 (0.92–1.07)
750 mg/day	1.09 (0.99–1.20)	1.02 (0.86–1.22)	1.05 (0.93–1.17)
P-linearity [3]	0.17	0.95	0.06
P-nonlinearity [4]	0.006	0.16	< 0.001
P-overall [5]	0.008	0.38	< 0.001
**Cardiometabolic disease**			
150 mg/day	1 (reference)	1 (reference)	1 (reference)
300 mg/day	1.10 (1.03–1.18)	0.97 (0.85–1.11)	0.90 (0.86–0.95)
450 mg/day	1.16 (1.04–1.30)	1.03 (0.85–1.25)	0.89 (0.83–0.97)
600 mg/day	1.19 (1.04–1.36)	1.16 (0.91–1.48)	0.93 (0.82–1.06)
750 mg/day	1.18 (1.00–1.40)	1.33 (0.97–1.83)	0.98 (0.80–1.19)
P-linearity [3]	0.08	0.11	0.008
P-nonlinearity [4]	0.04	0.07	0.005
P-overall [5]	0.03	0.06	< 0.001
	No. of eggs consumed per week		
**All causes**			
1 egg/week	1 (reference)	1 (reference)	1 (reference)
3 eggs/week	1.03 (1.00–1.06)	0.99 (0.94–1.04)	0.96 (0.93–0.99)
5 eggs/week	1.05 (1.00–1.10)	0.99 (0.92–1.06)	0.96 (0.92–1.00)
7 eggs/week	1.06 (1.01–1.11)	1.00 (0.93–1.07)	0.99 (0.95–1.03)
10 eggs/week	1.06 (1.00–1.12)	1.02 (0.93–1.12)	1.04 (0.99–1.10)
P-linearity [3]	0.07	0.66	0.25
P-nonlinearity [4]	0.18	0.44	< 0.001
P-overall [5]	0.08	0.68	0.002
**Cardiometabolic disease**			
1 egg/week	1 (reference)	1 (reference)	1 (reference)
3 eggs/week	0.98 (0.94–1.03)	0.93 (0.85–1.03)	0.96 (0.91–1.01)
5 eggs/week	0.98 (0.91–1.06)	0.94 (0.82–1.07)	0.95 (0.89–1.02)
7 eggs/week	1.00 (0.92–1.09)	0.99 (0.86–1.14)	0.98 (0.91–1.05)
10 eggs/week	1.05 (0.96–1.14)	1.14 (0.97–1.35)	1.03 (0.94–1.13)
P-linearity [3]	0.14	0.14	0.72
P-nonlinearity [4]	0.18	0.03	0.05
P-overall [5]	0.14	0.03	0.13

Abbreviations: CI, confidence interval; HR, hazard ratio.

[1] Adjusted for age, sex, education, annual income, marital status, total energy intake, smoking status, smoking pack-years, alcohol consumption, physical activity level, body mass index, healthy eating index, history of diabetes, hypertension, dyslipidemia, and hormone replacement therapy (for women only).

[2] Intake of refined carbohydrate was further adjusted for in the Chinese population.

[3] *P*-linearity values were obtained from Cox proportional hazard regression models with the exposure modeled as a linear term.

[4] *P*-nonlinearity values were obtained from Cox proportional hazard regression models with the exposure modeled as both cubic spline and linear terms.

[5] *P*-overall values were obtained for Cox proportional hazard regression models with the exposure modeled as a cubic spline term.

Table S4. All-cause and cardiometabolic mortality in relation to dietary cholesterol intake: sequential adjustments of covariates and dietary factors

600 mg/day vs. 150 mg/day	**Blacks (*n* = 47,789)**	**Whites (*n* = 20,360)**	**Chinese (*n* = 134,280)**

	HR (95% CI)	HR (95% CI)	HR (95% CI) [1]
	All-cause mortality		
Model 1 [2]	1.23 (1.14–1.32)	1.15 (1.03–1.28)	1.14 (1.07–1.22)
Model 2 [3]	1.21 (1.13–1.30)	1.11 (0.99–1.25)	1.12 (1.05–1.19)
Model 3 (main results) [4]	1.13 (1.05–1.21)	1.00 (0.89–1.12)	1.02 (0.95–1.09)
Model 3 + individual nutrients [5]			
Saturated fat	1.21 (1.11–1.31)	1.14 (0.99–1.30)	1.07 (0.99–1.15)
Carbohydrates	1.12 (1.04–1.21)	0.99 (0.88–1.11)	1.05 (0.97–1.13)
Protein	1.19 (1.09–1.31)	1.11 (0.96–1.28)	1.07 (1.00–1.16)
Fiber	1.13 (1.05–1.21)	1.01 (0.90–1.13)	1.09 (1.01–1.17)
Sodium	1.14 (1.05–1.24)	1.07 (0.94–1.23)	1.10 (1.02–1.18)
Model 3 + cholesterol-containing foods [5]			
Eggs	1.09 (0.99–1.21)	0.94 (0.79–1.12)	0.72 (0.61–0.85)
Red meat	1.11 (1.03–1.20)	1.01 (0.89–1.14)	1.07 (0.99–1.15)
Poultry	1.15 (1.06–1.24)	0.98 (0.87–1.10)	1.08 (1.00–1.16)
Fish	1.12 (1.04–1.21)	1.00 (0.89–1.13)	1.09 (1.01–1.17)
Shellfish	1.14 (1.06–1.23)	1.03 (0.92–1.15)	1.10 (1.02–1.18)
Dairy products	1.13 (1.05–1.22)	1.00 (0.90–1.12)	1.07 (1.00–1.16)
	Cardiometabolic mortality		
Model 1 [2]	1.31 (1.14–1.49)	1.44 (1.15–1.80)	1.06 (0.94–1.19)
Model 2 [3]	1.34 (1.19–1.50)	1.36 (1.12–1.66)	1.06 (0.95–1.18)
Model 3 (main results) [4]	1.21 (1.08–1.36)	1.12 (0.92–1.36)	0.94 (0.85–1.05)
Model 3 + individual nutrients [5]			
Saturated fat	1.36 (1.19–1.56)	1.44 (1.15–1.80)	1.14 (1.02–1.28)
Carbohydrates	1.22 (1.08–1.38)	1.15 (0.93–1.41)	1.01 (0.89–1.14)
Protein	1.18 (1.02–1.37)	1.26 (0.99–1.61)	1.04 (0.92–1.17)
Fiber	1.21 (1.08–1.36)	1.13 (0.93–1.38)	1.08 (0.96–1.22)
Sodium	1.14 (1.00–1.30)	1.23 (0.98–1.55)	1.10 (0.97–1.24)
Model 3 + cholesterol-containing foods [5]			
Eggs	1.18 (1.00–1.39)	0.95 (0.69–1.31)	0.59 (0.45–0.77)
Red meat	1.17 (1.04–1.33)	1.12 (0.91–1.38)	1.05 (0.94–1.19)
Poultry	1.24 (1.10–1.40)	1.07 (0.87–1.31)	1.06 (0.95–1.20)
Fish	1.18 (1.04–1.33)	1.13 (0.93–1.39)	1.09 (0.97–1.23)
Shellfish	1.21 (1.07–1.37)	1.14 (0.93–1.39)	1.09 (0.96–1.26)
Dairy products	1.21 (1.08–1.36)	1.12 (0.92–1.36)	1.07 (0.95–1.20)

Abbreviations: CI, confidence interval; HR, hazard ratio.

[1] Intake of refined carbohydrate was further adjusted for in the Chinese population.

[2] Adjusted for age, sex, education, annual income, marital status, and total energy intake.

[3] Adjusted for age, sex, education, annual income, marital status, total energy intake, smoking status, smoking pack-years, alcohol consumption, physical activity level, and hormone replacement therapy (for women only).

[4] Adjusted for age, sex, education, annual income, marital status, total energy intake, smoking status, smoking pack-years, alcohol consumption, physical activity level, hormone replacement therapy (for women only), body mass index, healthy eating index, and history of diabetes, hypertension, dyslipidemia, coronary heart disease, and stroke.

[5] Excluding the healthy eating index from each model.

Table S5. All-cause and cardiometabolic mortality in relation to eggs intake: sequential adjustments of covariates and dietary factors.

10 eggs/week vs. 1 egg/week	Blacks (*n* = 47,789)	Whites (*n* = 20,360)	Chinese (*n* = 134,280)
	HR (95% CI)	HR (95% CI)	HR (95% CI) [1]
	All-cause mortality		

Model 1 [2]	1.15 (1.09–1.20)	1.14 (1.05–1.23)	1.10 (1.05–1.16)
Model 2 [3]	1.12 (1.07–1.17)	1.11 (1.03–1.20)	1.11 (1.06–1.17)
Model 3 (main results) [4]	1.07 (1.02–1.12)	1.05 (0.97–1.14)	1.06 (1.01–1.11)
Model 3 + individual nutrients [5]			
Dietary cholesterol	1.07 (1.01–1.14)	1.12 (1.00–1.26)	1.39 (1.25–1.54)
Saturated fat	1.08 (1.03–1.13)	1.07 (0.99–1.16)	1.07 (1.02–1.12)
Carbohydrates	1.07 (1.02–1.12)	1.05 (0.97–1.13)	1.06 (1.01–1.12)
Protein	1.07 (1.02–1.12)	1.06 (0.98–1.15)	1.07 (1.02–1.12)
Fiber	1.07 (1.02–1.12)	1.05 (0.97–1.14)	1.06 (1.01–1.11)
Sodium	1.07 (1.02–1.12)	1.07 (0.99–1.16)	1.09 (1.03–1.14)
Model 3 + cholesterol-containing foods [5]			
Red meat	1.07 (1.02–1.13)	1.05 (0.97–1.13)	1.05 (1.00–1.11)
Poultry	1.07 (1.02–1.12)	1.05 (0.97–1.14)	1.05 (1.00–1.10)
Fish	1.07 (1.02–1.12)	1.05 (0.97–1.14)	1.06 (1.01–1.11)
Shellfish	1.07 (1.02–1.12)	1.05 (0.97–1.13)	1.06 (1.01–1.11)
Dairy products	1.07 (1.02–1.12)	1.05 (0.97–1.13)	1.06 (1.01–1.12)
Cardiometabolic mortality			
Model 1 [2]	1.15 (1.07–1.24)	1.27 (1.11–1.45)	1.09 (1.01–1.18)
Model 2 [3]	1.13 (1.05–1.21)	1.24 (1.09–1.41)	1.10 (1.02–1.18)
Model 3 (main results) [4]	1.07 (0.99–1.15)	1.14 (1.00–1.30)	1.03 (0.95–1.11)
Model 3 + individual nutrients [5]			
Dietary cholesterol	1.07 (1.01–1.14)	1.12 (1.00–1.26)	1.39 (1.25–1.54)
Saturated fat	1.07 (0.99–1.16)	1.18 (1.03–1.35)	1.06 (0.98–1.15)
Carbohydrates	1.07 (0.99–1.15)	1.14 (1.00–1.30)	1.05 (0.97–1.13)
Protein	1.06 (0.99–1.15)	1.14 (1.00–1.31)	1.05 (0.97–1.13)
Fiber	1.07 (0.99–1.15)	1.14 (1.00–1.30)	1.03 (0.95–1.11)
Sodium	1.05 (0.98–1.14)	1.15 (1.01–1.32)	1.08 (1.00–1.17)
Model 3 + cholesterol-containing foods [5]			
Red meat	1.08 (1.00–1.17)	1.14 (1.00–1.30)	1.02 (0.95–1.10)
Poultry	1.07 (0.99–1.15)	1.14 (1.00–1.30)	1.02 (0.95–1.10)
Fish	1.07 (1.00–1.16)	1.14 (1.00–1.30)	1.03 (0.95–1.11)
Shellfish	1.07 (0.99–1.15)	1.13 (0.99–1.29)	1.03 (0.95–1.11)
Dairy products	1.07 (0.99–1.15)	1.13 (0.99–1.29)	1.05 (0.97–1.13)

Abbreviations: CI, confidence interval; HR, hazard ratio.
[1] Intake of refined carbohydrate was further adjusted for in the Chinese population.
[2] Adjusted for age, sex, education, annual income, marital status, and total energy intake.
[3] Adjusted for age, sex, education, annual income, marital status, total energy intake, smoking status, smoking pack-years, alcohol consumption, physical activity level, and hormone replacement therapy (for women only).
[4] Adjusted for age, sex, education, annual income, marital status, total energy intake, smoking status, smoking pack-years, alcohol consumption, physical activity level, hormone replacement therapy (for women only), body mass index, healthy eating index, and history of diabetes, hypertension, dyslipidemia, coronary heart disease, and stroke.
[5] Excluding the healthy eating index from each model.

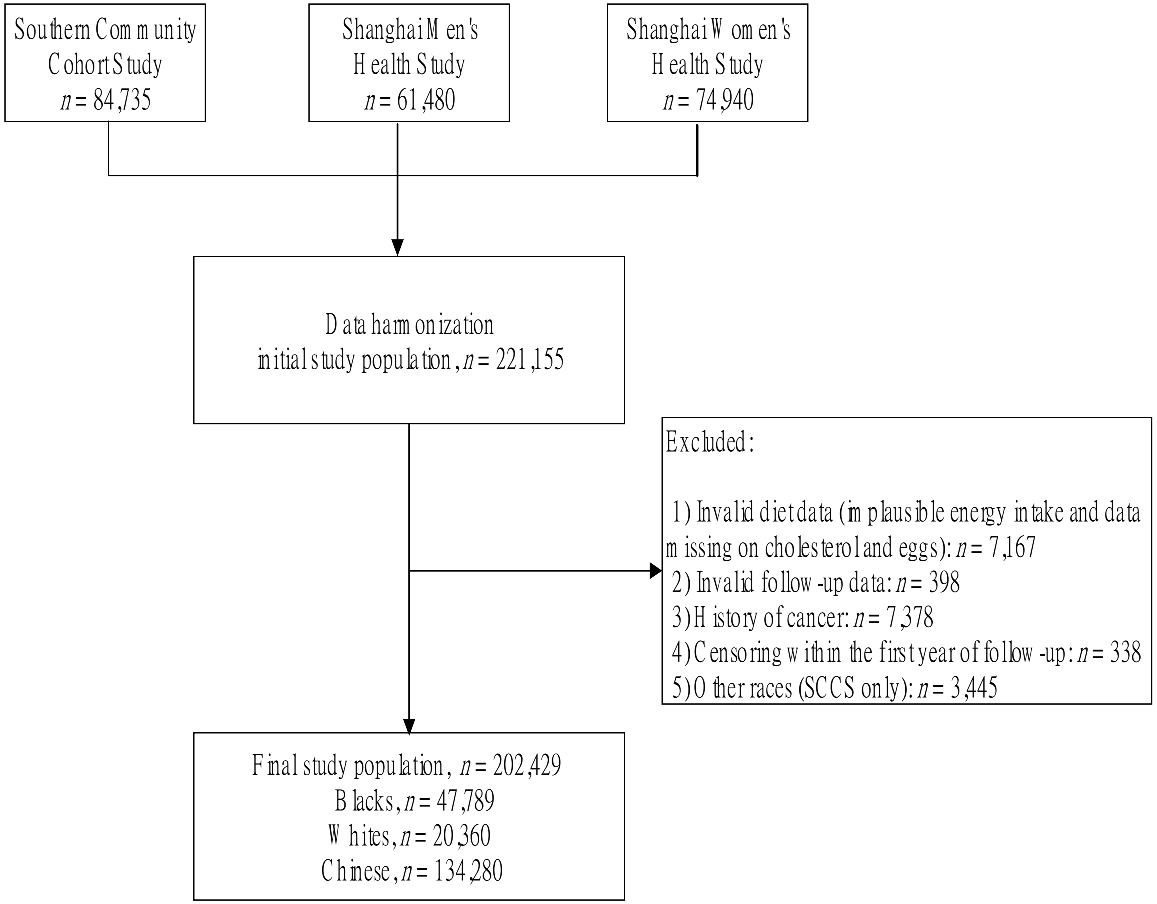

Figure S1. Flowchart of participant selection and exclusion in three cohorts.

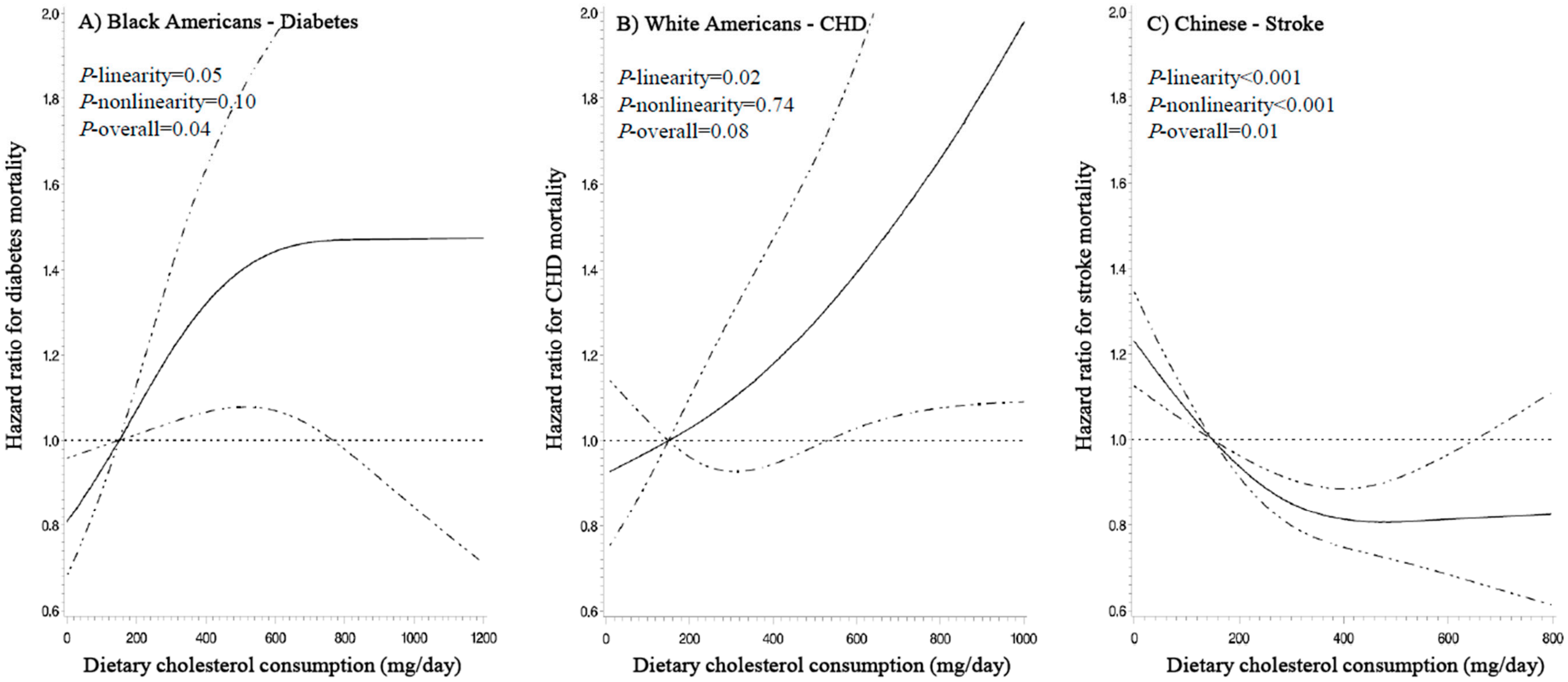

Figure S2. Dose-response relationships of dietary cholesterol intake with diabetes mortality among Blacks, CHD mortality among Whites, and stroke mortality among Chinese. **HRs (solid line) and 95% CIs (dashed line) were adjusted for age, sex, education, annual income, marital status, total energy intake, smoking status, smoking pack-years, alcohol consumption, physical activity level, body mass index, healthy eating index, history of diabetes, hypertension, dyslipidemia, coronary heart disease, and stroke, and hormone replacement therapy (for women only).** Intake of refined carbohydrate was further adjusted for in the Chinese population. To minimize potential effects of extreme values, participants with the top 1% of cholesterol intake were excluded from the analysis. 150 mg/day was set as the reference and three knots were fitted at the 5th, 50th, and 95th percentiles. *P*-nonlinearity values were 0.10 for diabetes mortality in Blacks, 0.74 for coronary heart disease mortality in Whites, and <0.001 for stroke mortality in Chinese. Abbreviations: CHD, coronary heart disease, CI, confidence interval; HR, hazard ratio.

.

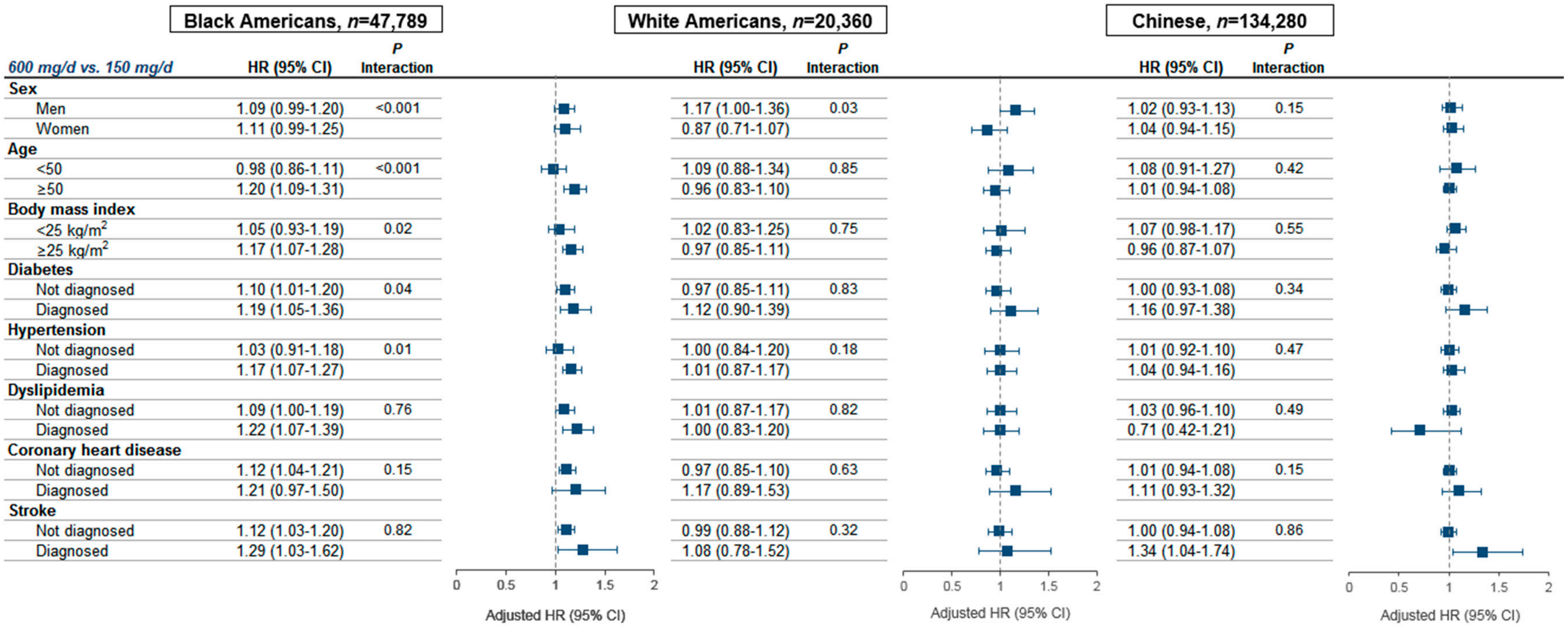

Figure S3. All-cause mortality in relation to dietary cholesterol intake: subgroup analyses. HRs (95% CIs) represent 600 mg/day vs. 150 mg/day of dietary cholesterol consumption. Estimates were adjusted for age, sex, education, annual income, marital status, total energy intake, smoking status, smoking pack-years, alcohol consumption, physical activity level, body mass index, healthy eating index, history of diabetes, hypertension, dyslipidemia, coronary heart disease, and stroke, and hormone replacement therapy (for women only). Intake of refined carbohydrate was further adjusted for in the Chinese population. Abbreviations: CI, confidence interval; HR, hazard ratio.

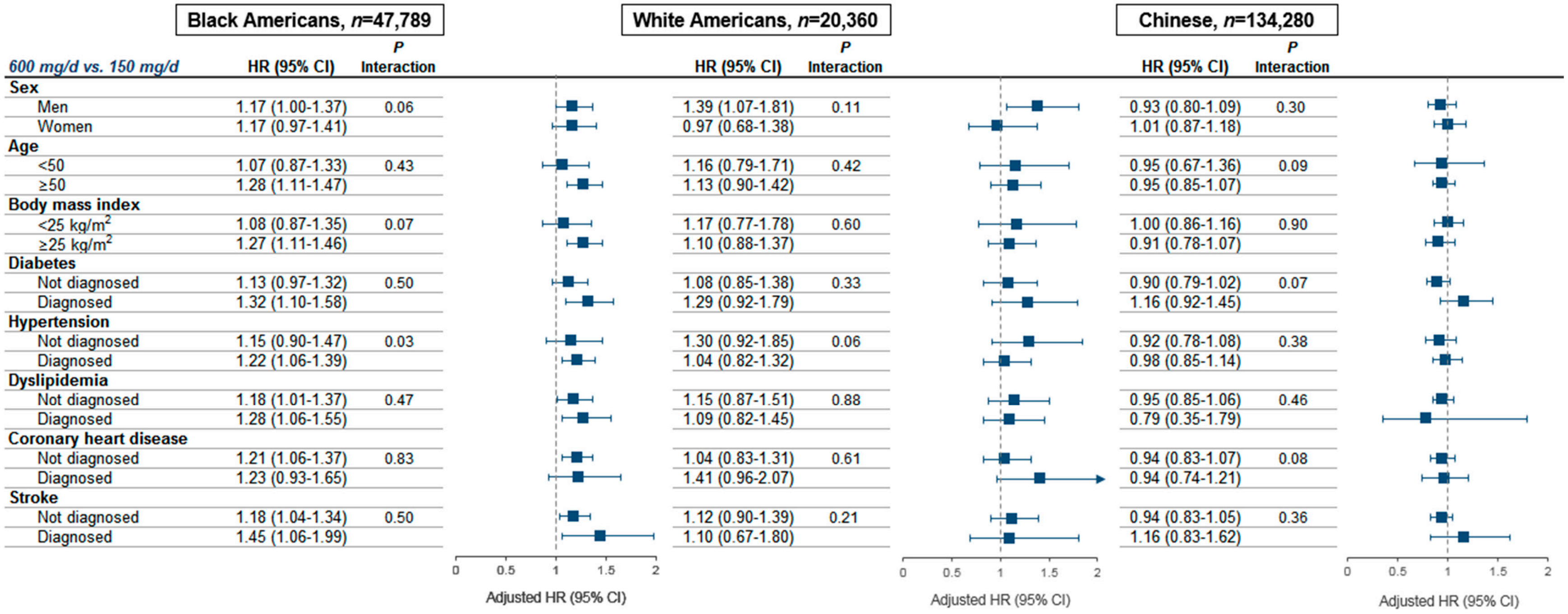

Figure S4. Cardiometabolic mortality in relation to dietary cholesterol intake: subgroup analyses**.** HRs (95% CIs) represent 600 mg/day vs. 150 mg/day of dietary cholesterol consumption. Estimates were adjusted for age, sex, education, annual income, marital status, total energy intake, smoking status, smoking pack-years, alcohol consumption, physical activity level, body mass index, healthy eating index, history of diabetes, hypertension, dyslipidemia, coronary heart disease, and stroke, and hormone replacement therapy (for women only). Intake of refined carbohydrate was further adjusted for in the Chinese population. Abbreviations: CI, confidence interval; HR, hazard ratio.

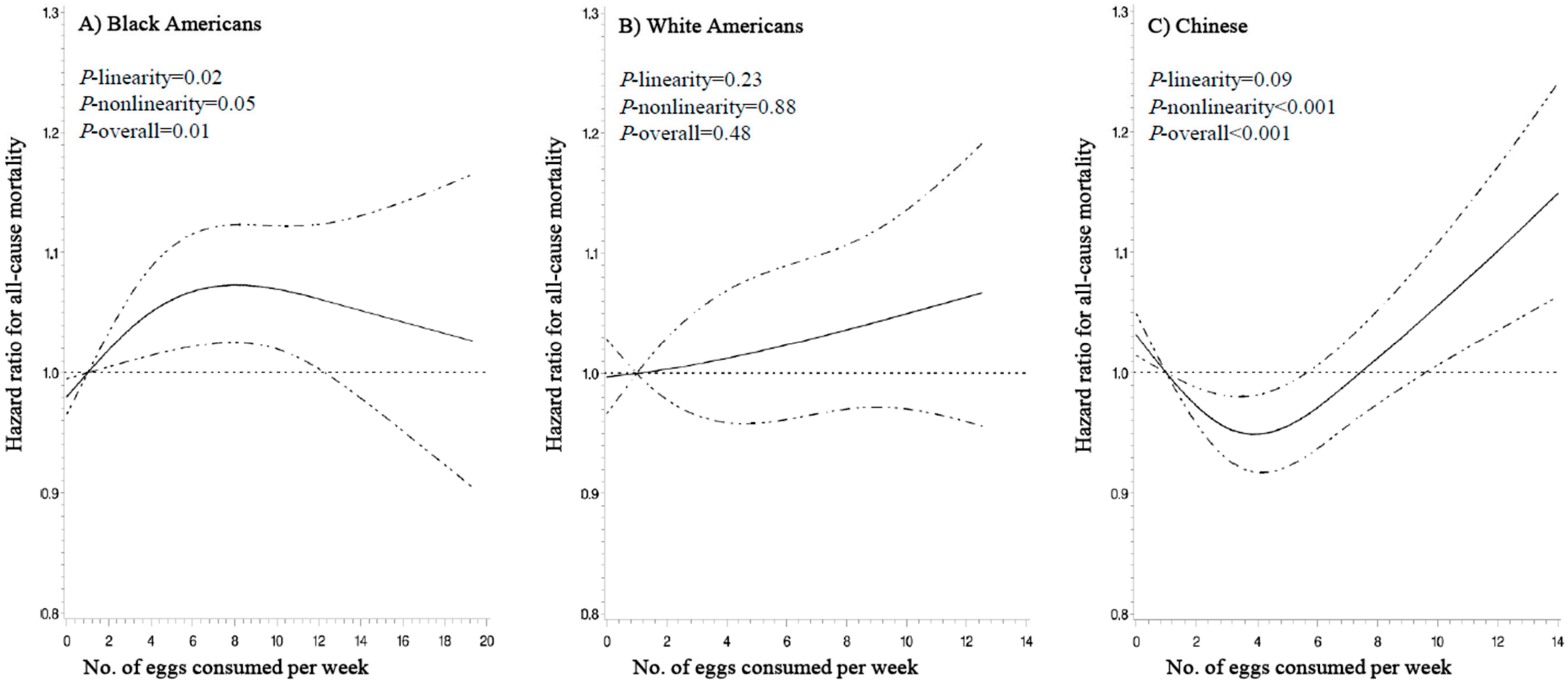

Figure S5. Dose-response relationships of egg intake with all-cause mortality**.** HRs (solid line) and 95% CIs (dashed line) were adjusted for age, sex, education, annual income, marital status, total energy intake, smoking status, smoking pack-years, alcohol consumption, physical activity level, body mass index, healthy eating index, history of diabetes, hypertension, dyslipidemia, coronary heart disease, and stroke, and hormone replacement therapy (for women only). Intake of refined carbohydrate was further adjusted for in the Chinese population. To minimize potential effects of extreme values, participants with the top 1% of egg intake were excluded from the analysis. One egg consumed per week was set as the reference, and three knot positions were fitted at the 5th, 50th, and 95th percentiles. *P*-nonlinearity values for all-cause mortality were 0.05 in Blacks, 0.88 in Whites, and <0.001 in Chinese, respectively. Abbreviations: CI, confidence interval; HR, hazard ratio.

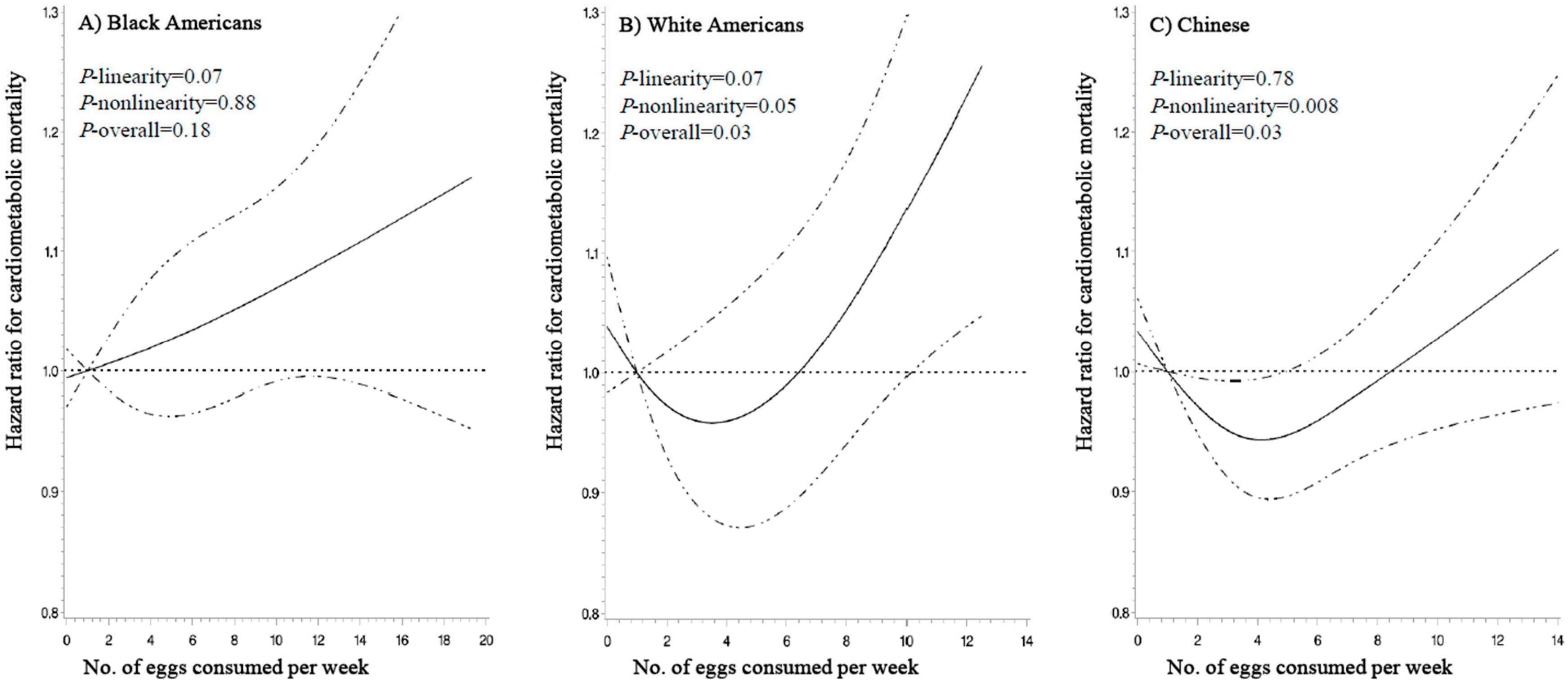

Figure S6. Dose-response relationships of egg intake with cardiometabolic mortality**.** HRs (solid line) and 95% CIs (dashed line) were adjusted for age, sex, education, annual income, marital status, total energy intake, smoking status, smoking pack-years, alcohol consumption, physical activity level, body mass index, healthy eating index, history of diabetes, hypertension, dyslipidemia, coronary heart disease, and stroke, and hormone replacement therapy (for women only). Intake of refined carbohydrate was further adjusted for in the Chinese population. To minimize potential effects of extreme values, participants with the top 1% of egg intake were excluded from the analysis. One egg consumed per week was set as the reference, and three knot positions were fitted at the 5th, 50th, and 95th percentiles. *P*-nonlinearity values for cardiometabolic mortality were 0.88, 0.05, and 0.008, respectively. Abbreviations: CI, confidence interval; HR, hazard ratio.
